# 浆细胞白血病诊断与治疗中国专家共识（2025年版）

**DOI:** 10.3760/cma.j.cn121090-20250618-00283

**Published:** 2025-08

**Authors:** 中国临床肿瘤学会（CSCO）骨髓瘤专家委员会 中国抗癌协会（CACA）血液肿瘤专业委员会骨髓瘤与浆细胞疾病学组 中华医学会血液学分会浆细胞疾病学组

## Abstract

浆细胞白血病（PCL）是一种少见且侵袭性极高的浆细胞恶性肿瘤，临床进展迅速，预后极差。本共识旨在为临床医师提供PCL诊断标准、风险评估、治疗策略（包括初始治疗、造血干细胞移植和新型药物应用）及随访管理的系统建议，以规范诊疗流程，提高患者的生存率与生活质量。

## Abstract (en)

Plasma cell leukemia（PCL）is a rare and highly aggressive form of plasma cell dyscrasia, distinguished by its rapid clinical course and dismal prognosis. This guideline is intended to offer clinicians evidence-based, comprehensive recommendations encompassing diagnostic criteria, risk stratification, therapeutic approaches—including frontline treatment, hematopoietic stem cell transplantation, and the incorporation of novel therapeutic agents—as well as protocols for long-term follow-up. The overarching objective is to promote standardized clinical management and ultimately enhance both survival outcomes and quality of life for patients diagnosed with PCL.

浆细胞白血病（plasma cell leukemia，PCL）是浆细胞恶性肿瘤中侵袭性最强的类型。现有数据显示，尽管采用新型药物治疗，不适合接受自体造血干细胞移植（auto-HSCT）的原发性PCL（pPCL）患者的中位总生存（OS）期仅约2年，而适合移植者通过auto-HSCT可将OS期延长至3年或更久。PCL近年来取得的治疗进展与多发性骨髓瘤（MM）仍存在显著差距。随着新型治疗策略的不断探索，这一临床难题有望获得突破。为规范我国PCL诊疗实践，提升临床医师诊治水平，中国临床肿瘤学会（CSCO）骨髓瘤专家委员会、中国抗癌协会（CACA）血液肿瘤专业委员会、中华医学会血液学分会浆细胞疾病学组组织国内权威专家通过多轮研讨、论证，最终形成本版专家共识。

一、定义、临床表现及生物学特征

根据是否存在MM前驱病史，PCL可分为pPCL和继发性PCL（secondary PCL，sPCL）两类。2021年国际骨髓瘤工作组（IMWG）更新了pPCL的诊断标准，将外周血循环浆细胞（circulating plasma cell，CPC）阈值从20％下调至5％。更新后的pPCL定义为在满足MM诊断标准的基础上，外周血CPC≥5％的恶性浆细胞疾病，约占新诊断MM（newly diagnosed MM, NDMM）患者的6％[1]。sPCL则发生于既往确诊的MM患者中，多见于多线治疗后的复发难治性MM（relapsed/refractory MM，RRMM）患者，占RRMM病例的1％，其中位生存期仅1～4个月[2]–[4]。需要说明的是，目前sPCL的CPC诊断阈值尚未完全统一，但越来越多的研究采用5％作为临界值[2],[5]–[6]。

pPCL的中位发病年龄为52～65岁，较MM患者发病年龄低约10岁[7]。在其M蛋白类型中，轻链型和不分泌型更为常见。pPCL起病急，肿瘤负荷高，症状显著，常伴严重贫血、血小板减少、肾功能不全、高钙血症。pPCL患者的溶骨性病变较MM患者少见，但髓外浸润在pPCL中更为普遍。sPCL同样表现为高度侵袭性且肿瘤负荷高[4]。

pPCL细胞在形态学、免疫表型和遗传学特征上与典型MM细胞有所不同[8]–[12]。形态学方面，pPCL细胞常表现为淋巴细胞样或浆母细胞样形态。免疫表型方面，pPCL细胞中CD56表达显著下调，而CD20、CD44、CD45和CD19的表达水平升高，同时CD11a、CD27、CD28、CD81、CD71、CD9、HLA-DR和CD117的表达降低。上述黏附分子表达失衡导致浆细胞与骨髓微环境的相互作用减弱，因此肿瘤细胞更易进入外周血循环。从细胞遗传学特征来看，pPCL亚二倍体常见，同时t（11;14）、t（14;16）等染色体易位较常见。此外，高危遗传学异常（如TP53缺失或突变、1号染色体异常、13q缺失）的发生率更高。基因组分析表明，pPCL的基因具有高度不稳定性，表现为KRAS/NRAS/BRAF等基因突变频发、TP53等抑癌基因失活现象显著，且常伴随APOBEC相关突变[13]。相比之下，sPCL的免疫表型、细胞遗传学、基因组学与RRMM更为相似，被认为是MM疾病终末期的表现[14]。

二、诊断所需的检测项目

PCL的诊断应建立在MM的基础上，因此所有新诊断患者都应进行MM相关检查。表1列出了诊断PCL必备的检查项目[15]。

**表1:** 诊断浆细胞白血病（PCL）时必需的检查类别及项目

类别	检查/评估项目
病史和体格检查	完整的病史采集（包括骨痛、乏力、发热等）； 体格检查（包括皮下包块，肝、脾、淋巴结肿大，神经系统症状等）； 体能状态评估； 如果年龄≥65岁，依据国际骨髓瘤工作组的老年评分系统进行衰弱评分
尿液检查	24 h尿蛋白定量、24 h尿蛋白电泳、24 h尿免疫固定电泳
血液学检查	全血细胞计数及分类； 外周血涂片和（或）多参数流式细胞术^a^检测外周血浆细胞； 血清蛋白电泳、血清免疫固定电泳、血清游离轻链检测； 血生化：至少包括白蛋白、球蛋白、肝功能、碱性磷酸酶、乳酸脱氢酶、血肌酐及血钙；氨基末端脑钠肽前体（NT-proBNP）或脑钠肽（BNP）、心肌肌钙蛋白I（cTnI）； β_2_-微球蛋白； 乙型肝炎标志物
影像学检查	低剂量全身CT（优于传统骨骼X线）或全身磁共振成像（WB-MRI）^b^或PET/CT
骨髓检查	骨髓涂片、骨髓多参数流式细胞术^a^、二代测序、染色体核型、荧光原位杂交^c^；骨髓活检及免疫组织化学染色
其他检查	怀疑继发淀粉样变性，进行淀粉样变性相关检测；怀疑中枢神经系统侵犯时进行腰椎穿刺（存在神经系统症状时必须进行腰椎穿刺，高循环浆细胞患者建议进行腰椎穿刺；初始治疗清除循环浆细胞后操作，避免肿瘤播散）

**注** ^a^怀疑PCL的患者应进行外周血多参数流式细胞术检测，以CD45^−/dim^CD38^+^细胞设门，同时应包括CD138、CD56、CD19、CD27、CD20、CD81、CD117、HLA-DR及胞质κ和λ、BCMA、GPRC5D；^b^WB-MRI尤其是弥散加权MRI对于识别髓外病灶具有较高的灵敏性；^c^荧光原位杂交检测前建议用CD138磁珠分选肿瘤细胞，检测位点建议包括：IgH重排、17p缺失、13q14缺失、1q21增加/扩增、1p32缺失、Myc易位，若IgH重排阳性，则进一步检测t（4;14）、t（11;14）、t（14;16）、t（14;20）

专家共识：①PCL的诊断应在MM基础上进行，所有新诊断患者均需完成MM相关检查。②怀疑PCL患者应进行外周血涂片检查，并至少计数200个有核细胞。多参数流式细胞术有助于识别形态学不典型的异常浆细胞，对疑似PCL患者均应进行外周血多参数流式细胞术检测。③PCL的诊断标准为：在满足MM诊断标准的基础上，外周血CPC比例≥5％的恶性浆细胞疾病。根据是否存在MM前驱病史，PCL可分为pPCL和sPCL。

三、预后评估与危险分层

所有pPCL均应视为超高危的浆细胞肿瘤。目前pPCL尚未建立统一的标准化分期及危险度分层系统，建议参考MM的风险评估框架。有研究提出简化评分模型，纳入三项危险因素：年龄>60岁、血小板计数<100×10⁹/L以及外周血CPC>20×10⁹/L。根据该模型，无危险因素患者的中位OS期为46个月，含1项危险因素者OS期降至27个月，而具有2项及以上危险因素者OS期仅12个月[16]。研究显示，伴有高危细胞遗传学异常的pPCL患者预后更差，但t（11;14）阳性PCL患者的预后优于t（11;14）阴性者[17]。

专家共识：①pPCL目前缺乏统一的预后分期系统，临床评估需综合以下因素：患者年龄、CPC水平、血小板计数及细胞遗传学异常。②值得注意的是，携带t（11;14）染色体易位的pPCL患者通常预后较好。

四、初治pPCL的治疗

1. 初治pPCL的治疗原则：由于pPCL相对少见，目前尚缺乏大规模前瞻性临床试验，因此其标准治疗尚未确定。专家委员会认为，pPCL的总体治疗策略包括以下三点：①采用不同作用机制的多种药物联合治疗，并持续治疗；②以根除所有肿瘤克隆为目标，尽可能实现并维持骨髓内外微小残留病（minimal residual disease，MRD）阴性状态；③鉴于pPCL当前疗效有限，建议优先入组符合条件的临床试验。

2. 适合移植的初治pPCL的治疗：

（1）移植前的诱导治疗：在pPCL中，诱导治疗不仅用于快速控制高肿瘤负荷，更应力求实现深度缓解，甚至达到MRD阴性，从而为后续的移植和长期疾病控制奠定基础。鉴于pPCL临床进展迅速，且对标准治疗方案反应不佳，建议尽早采用强化诱导方案，推荐以CD38单克隆抗体、蛋白酶体抑制剂、免疫调节剂及地塞米松四药为基础的方案，可以联合细胞毒性药物方案（如OPTIMUM/MUKnine研究[18]）。此外，有研究表明，采用卡非佐米方案治疗pPCL患者可能带来显著益处（如EMN12/HOVON-129研究[19]）。对于体能状态良好、年轻、高肿瘤负荷患者，可在新药基础上联合多种细胞毒性药物，CD38单克隆抗体+VRD/KRD-PACE（硼替佐米/卡非佐米+来那度胺+地塞米松+顺铂+多柔比星+环磷酰胺+依托泊苷）或者CD38单克隆抗体-DECP（地塞米松+依托泊苷+环磷酰胺+顺铂）[20]。

（2）造血干细胞移植：作为整体治疗的基石，auto-HSCT可增加pPCL患者治疗深度并延长生存期[21]。串联移植（tandem transplantation）指在第一次auto-HSCT后的6个月内进行计划中的第2次auto-HSCT。既往研究表明，pPCL患者可能从串联移植中获益[22]–[23]。建议患者在首次动员后尽量采集满足两次auto-HSCT所需的造血干细胞数量，建议第一次移植后6个月内进行串联移植。两次移植采用的预处理方案通常为大剂量美法仑。异基因造血干细胞移植（allo-HSCT）治疗的复发率低于auto-HSCT，但治疗风险和非复发死亡率较高[24]，故作为PCL的一线治疗仍存在争议。但对于<50岁、体能状态良好且有合适供者的患者，也可考虑进行清髓性allo-HSCT或auto-HSCT序贯减低强度非清髓性allo-HSCT；对于>50岁，体能状态良好且有合适供者的患者，可考虑auto-HSCT序贯减低强度非清髓性allo-HSCT。研究表明，诱导治疗后未达到完全缓解（CR）、体能状态良好且有合适供者的患者，从auto-HSCT序贯减低强度非清髓性allo-HSCT中获益较大[22]。有研究评估了初诊适合移植的pPCL患者应用auto-HSCT联合自体靶向BCMA的CAR-T细胞输注的疗效。初步结果显示，CAR-T细胞治疗在pPCL治疗中展现出巨大潜力，为高风险疾病带来了新希望[3]。

（3）巩固治疗：串联auto-HSCT后，若仍未达到CR和（或）MRD阴性，建议采用原诱导方案继续巩固治疗2个疗程；若2个疗程后仍未达到CR和（或）MRD阴性，可延长至4个疗程。对于已达到CR和（或）MRD阴性的患者，可考虑不进行巩固治疗。若患者未接受串联移植，则建议采用原诱导方案继续巩固治疗2～4个疗程[24]。

（4）维持治疗：推荐采用免疫调节剂、蛋白酶体抑制剂和（或）CD38单克隆抗体为基础的两药或三药方案进行维持治疗，持续治疗直至疾病进展或不能耐受。

专家共识：适合移植的pPCL治疗：①符合条件的患者优先纳入临床试验，尤其是包含CAR-T细胞治疗及双特异性抗体的临床试验。②在pPCL移植前，推荐采用含CD38单克隆抗体、蛋白酶体抑制剂、免疫调节剂及地塞米松的四药联合方案作为诱导治疗。具体方案包括：CD38单克隆抗体+VRd/KRd。对于体能状态良好、年轻且肿瘤负荷较高者，可使用CD38单克隆抗体+VRD/KRD-PACE或CD38单克隆抗体+DECP。③造血干细胞移植：<50岁，有合适供者：串联auto-HSCT或清髓性allo-HSCT或auto-HSCT序贯减低强度非清髓性allo-HSCT。<50岁，无合适供者：串联auto-HSCT。>50岁，有合适供者：串联auto-HSCT或auto-HSCT序贯减低强度非清髓性allo-HSCT。>50岁，无合适供者：串联auto-HSCT。④巩固治疗：若患者未接受串联移植或串联移植后未达到CR或MRD阴性，建议沿用原诱导方案巩固治疗2～4个疗程。⑤维持治疗：推荐应用含免疫调节剂、蛋白酶体抑制剂和（或）CD38单克隆抗体的两药或三药方案进行维持治疗，建议持续治疗直至疾病进展或不能耐受。⑥pPCL具有较高的中枢神经系统侵犯风险。如患者出现神经系统症状，应进行诊断性腰椎穿刺。为了避免肿瘤细胞播散至中枢神经系统，腰椎穿刺最好在初始治疗后待浆细胞减少或消失后再进行。

3. 不适合移植的初治pPCL的治疗：对于不适合移植的pPCL患者，首选临床试验。有临床试验正在探索CAR-T细胞治疗初诊不适合移植的pPCL患者。若无临床试验可选，需根据IMWG虚弱程度分级（GA评分[25]）进行体能状态评估，并制定个体化治疗方案。对于体能状态良好的患者，优先推荐以CD38单克隆抗体为基础的四药联合方案（联合蛋白酶体抑制剂、免疫调节剂及地塞米松）；针对虚弱患者，则采用CD38单克隆抗体联合蛋白酶体抑制剂或免疫调节剂的三药方案（含地塞米松）。建议持续治疗至疾病进展或出现不可耐受的不良反应。

专家共识：①优先考虑入组临床试验，尤其是包含CAR-T细胞治疗和双特异性抗体的临床试验。②推荐所有不适合移植的pPCL患者先行老年体能状态评估；对于体能状态良好的患者，推荐采用CD38单克隆抗体联合蛋白酶体抑制剂、免疫调节剂及地塞米松的四药联合方案持续治疗；对于体能状态较差或合并基础疾病的体弱患者，建议采用以CD38单克隆抗体为基础的三药联合方案持续治疗。

五、复发/难治pPCL的治疗

复发/难治pPCL患者预后极差，当前尚无统一标准治疗方案。治疗策略应综合既往用药、缓解深度、复发时间、年龄及体能状态等因素。根据年龄和体能状态，推荐联合既往未应用过的药物（如新一代蛋白酶体抑制剂、免疫调节剂、CD38单克隆抗体等）治疗。对挽救性治疗有反应且符合移植标准的患者，建议进行allo-HSCT；不适合进行移植的患者建议继续接受治疗，直至疾病出现进展或患者无法耐受治疗。对于携带t（11;14）易位的患者，可以考虑采用BCL-2抑制剂进行单药或联合治疗[26]–[27]。此外，多项小队列回顾性研究证实CAR-T细胞治疗对复发难治pPCL患者有良好的疗效[28]–[29]。双特异性抗体作为即用型药物，在临床上可能有潜力控制pPCL快速进展。

专家共识：①推荐所有复发/难治pPCL患者优先考虑入组临床试验，尤其是免疫治疗的临床试验；②推荐复发/难治pPCL患者选择新一代药物或不同作用机制药物的联合方案。

六、sPCL的治疗

sPCL是MM演变至终末期的临床表现，常在既往多线治疗后发生，呈现肿瘤克隆高度不稳定、快速进展和耐药等特征，预后极差。目前尚无公认的标准治疗方案，可参考pPCL的方案。多项小队列研究报道，CAR-T细胞治疗对于sPCL患者有一定的疗效，整体有效率近70％[28],[30]。双特异性抗体对于治疗sPCL患者也具有一定的潜力，但疗效常难以持续[31]–[32]。

专家共识：①推荐sPCL患者优先考虑入组临床试验，尤其是免疫治疗的临床试验；②推荐sPCL患者选择未应用过的新一代药物或不同作用机制药物的联合方案。

七、疗效评估

目前，PCL建议参照IMWG关于MM的疗效标准，同时结合外周血CPC检测判断疗效[7],[33]–[34]。然而，鉴于PCL具有白血病特性，轻链型和无分泌型多见，且常合并髓外病变，传统疗效评估标准无法全面反映PCL患者的治疗反应。因此，建议联合应用下一代流式细胞术（next-generation flow，NGF）或下一代测序技术（next-generation sequencing，NGS）动态监测PCL患者骨髓MRD。此外，针对PCL中CPC的独特生物学特征及临床意义，建议通过NGF动态监测外周残留病（peripheral residual disease，PRD）[35]。

专家共识：①PCL疗效评估参照IMWG的MM标准，同时结合外周血CPC检测；②推荐PCL患者动态监测骨髓和外周血肿瘤细胞。

八、小结与展望

PCL是一种预后极差的高度侵袭性浆细胞疾病，具有独特的生物学特征，包括基因组不稳定性、高增殖性及髓外病变发生率高等[2],[36]–[40]。2021年IMWG更新了诊断标准，将外周血中CPC比例≥5％作为pPCL的诊断阈值，显著扩大了患者范围[1]。当前，新型治疗手段尤其是蛋白酶体抑制剂、免疫调节剂和CD38单克隆抗体联合auto-HSCT的应用已显著改善了PCL患者的预后[18]–[19],[22]。未来的治疗策略将突破传统化疗和靶向治疗框架，更聚焦于免疫疗法。CAR-T细胞疗法正逐渐成为PCL的一线治疗，为PCL患者提供了新的治疗选择[29],[41]–[42]。随着诊断标准的更新、疾病生物学机制的深入研究以及治疗策略的持续优化，PCL患者有望获得更长的生存期与更好的生活质量。

## References

[1] Fernández de Larrea C, Kyle R, Rosiñol L (2021). Primary plasma cell leukemia: consensus definition by the International Myeloma Working Group according to peripheral blood plasma cell percentage[J]. Blood Cancer J.

[2] Singh S, Peshin S, Wertheim BC (2025). Outcomes and treatment patterns in primary and secondary plasma cell leukemia: insights from a large US cohort study[J]. Haematologica.

[3] Jurczyszyn A, Castillo JJ, Avivi I (2019). Secondary plasma cell leukemia: a multicenter retrospective study of 101 patients[J]. Leuk Lymphoma.

[4] Tessier C, LeBlanc R, Roy J (2024). Poor outcome despite modern treatments: A retrospective study of 99 patients with primary and secondary plasma cell leukemia[J]. Cancer Med.

[5] Guan J, Ma J, Chen B (2023). Clinical and cytogenetic characteristics of primary and secondary plasma cell leukemia under the new IMWG definition criteria: a retrospective study[J]. Hematology.

[6] Broughton M, Bhatta S, Sharma N (2024). Evaluation of characteristics and outcomes of patients with primary and secondary plasma cell leukemia treated with modern therapies[J]. Blood.

[7] Fernández de Larrea C, Kyle RA, Durie BG (2013). Plasma cell leukemia: consensus statement on diagnostic requirements, response criteria and treatment recommendations by the International Myeloma Working Group[J]. Leukemia.

[8] van de Donk NW, Lokhorst HM, Anderson KC (2012). How I treat plasma cell leukemia[J]. Blood.

[9] Jelinek T, Kryukov F, Rihova L (2015). Plasma cell leukemia: from biology to treatment[J]. Eur J Haematol.

[10] Bezdekova R, Jelinek T, Kralova R (2021). Necessity of flow cytometry assessment of circulating plasma cells and its connection with clinical characteristics of primary and secondary plasma cell leukaemia[J]. Br J Haematol.

[11] Neri A, Todoerti K, Lionetti M (2016). Primary plasma cell leukemia 2.0: advances in biology and clinical management[J]. Expert Rev Hematol.

[12] Musto P, Statuto T, Valvano L (2019). An update on biology, diagnosis and treatment of primary plasma cell leukemia[J]. Expert Rev Hematol.

[13] Maura F, Petljak M, Lionetti M (2018). Biological and prognostic impact of APOBEC-induced mutations in the spectrum of plasma cell dyscrasias and multiple myeloma cell lines[J]. Leukemia.

[14] Papadhimitriou SI, Terpos E, Liapis K (2022). The cytogenetic profile of primary and secondary plasma cell leukemia: etiopathogenetic perspectives, prognostic impact and clinical relevance to newly diagnosed multiple myeloma with differential circulating clonal plasma cells[J]. Biomedicines.

[15] van de Donk N (2023). How we manage newly diagnosed multiple myeloma with circulating tumor cells[J]. J Clin Oncol.

[16] Jurczyszyn A, Radocha J, Davila J (2018). Prognostic indicators in primary plasma cell leukaemia: a multicentre retrospective study of 117 patients[J]. Br J Haematol.

[17] Cazaubiel T, Leleu X, Perrot A (2022). Primary plasma cell leukemias displaying t(11;14) have specific genomic, transcriptional, and clinical features[J]. Blood.

[18] Kaiser MF, Hall A, Walker K (2023). Daratumumab, cyclophosphamide, bortezomib, lenalidomide, and dexamethasone as induction and extended consolidation improves outcome in ultra-high-risk multiple myeloma[J]. J Clin Oncol.

[19] van de Donk N, Minnema MC, van der Holt B (2023). Treatment of primary plasma cell leukaemia with carfilzomib and lenalidomide-based therapy (EMN12/HOVON-129): final analysis of a non-randomised, multicentre, phase 2 study[J]. Lancet Oncol.

[20] Lakshman A, Singh PP, Rajkumar SV (2018). Efficacy of VDT PACE-like regimens in treatment of relapsed/refractory multiple myeloma[J]. Am J Hematol.

[21] Mahindra A, Kalaycio ME, Vela-Ojeda J (2012). Hematopoietic cell transplantation for primary plasma cell leukemia: results from the Center for International Blood and Marrow Transplant Research[J]. Leukemia.

[22] Lawless S, Iacobelli S, Knelange NS (2023). Comparison of autologous and allogeneic hematopoietic cell transplantation strategies in patients with primary plasma cell leukemia, with dynamic prediction modeling[J]. Haematologica.

[23] Royer B, Minvielle S, Diouf M (2016). Bortezomib, doxorubicin, cyclophosphamide, dexamethasone induction followed by stem cell transplantation for primary plasma cell leukemia: a prospective phase II study of the Intergroupe Francophone du Myélome[J]. J Clin Oncol.

[24] Musto P, Engelhardt M, van de Donk N (2025). European Myeloma Network Group review and consensus statement on primary plasma cell leukemia[J]. Ann Oncol.

[25] Palumbo A, Bringhen S, Mateos MV (2015). Geriatric assessment predicts survival and toxicities in elderly myeloma patients: an International Myeloma Working Group report[J]. Blood.

[26] Jelinek T, Mihalyova J, Kascak M (2019). Single-agent venetoclax induces MRD-negative response in relapsed primary plasma cell leukemia with t(11;14)[J]. Am J Hematol.

[27] Nalghranyan S, Singh AP, Schinke C (2020). The combination of venetoclax, daratumumab and dexamethasone for the treatment of refractory primary plasma cell leukemia[J]. Am J Hematol.

[28] Fortuna GMG, Sidana S, Hovanky V (2024). Idecabtagene Vicleucel (ide-cel) chimeric antigen receptor T-cell for plasma cell leukemia (PCL): a multicenter experience[J]. Transplantation and Cellular Therapy.

[29] Li C, Cao W, Que Y (2021). A phase I study of anti-BCMA CAR T cell therapy in relapsed/refractory multiple myeloma and plasma cell leukemia[J]. Clin Transl Med.

[30] Guo Y, Hu K, Ke X (2023). PB2101: a prospective investigation into the timing and status of BCMA-CART in secondary plasma cell leukemia[J]. Hemasphere.

[31] Shrestha A, Thanendrarajan S, Bag A (2024). The efficacy of teclistamab in multiple myeloma patients with secondary plasma cell leukemia[J]. Blood.

[32] Mohan M, Monge J, Shah N (2024). Teclistamab in relapsed refractory multiple myeloma: multi-institutional real-world study[J]. Blood Cancer J.

[33] Kumar S, Paiva B, Anderson KC (2016). International Myeloma Working Group consensus criteria for response and minimal residual disease assessment in multiple myeloma[J]. Lancet Oncol.

[34] Tuazon SA, Holmberg LA, Nadeem O (2021). A clinical perspective on plasma cell leukemia; current status and future directions[J]. Blood Cancer J.

[35] Paiva B, Shi Q, Puig N (2025). Opportunities and challenges for MRD assessment in the clinical management of multiple myeloma[J]. Nat Rev Clin Oncol.

[36] Saba L, Landau KS, Liang H (2024). Real world analysis on the determinants of survival in primary plasma cell leukemia in the United States[J]. Leukemia.

[37] Yan W, Fan H, Xu J (2022). The clinical characteristics and prognosis of patients with primary plasma cell leukemia (pPCL) according to the new IMWG definition criteria[J]. Leuk Lymphoma.

[38] Cazaubiel T, Leleu X, Perrot A (2022). Primary plasma cell leukemias displaying t(11;14) have specific genomic, transcriptional, and clinical features[J]. Blood.

[39] Nandakumar B, Kumar SK, Dispenzieri A (2021). Clinical characteristics and outcomes of patients with primary plasma cell leukemia in the era of novel agent therapy[J]. Mayo Clin Proc.

[40] Tiedemann RE, Gonzalez-Paz N, Kyle RA (2008). Genetic aberrations and survival in plasma cell leukemia[J]. Leukemia.

[41] Sidana S, Patel KK, Peres LC (2025). Safety and efficacy of standard-of-care ciltacabtagene autoleucel for relapsed/refractory multiple myeloma[J]. Blood.

[42] Xu J, Zhang S, Yan W (2024). A phase 2 study of CART-ASCT-CART2 sandwich regimen treatment in patients with primary plasma cell leukemia[J]. Blood.

